# Camouflaging in an everyday social context: An interpersonal recall study

**DOI:** 10.1177/1362361321992641

**Published:** 2021-02-19

**Authors:** Julia Cook, Laura Crane, Laura Bourne, Laura Hull, William Mandy

**Affiliations:** University College London, UK

**Keywords:** adults, autism spectrum disorders, qualitative research, social cognition, social behaviour

## Abstract

Camouflaging is a social phenomenon operating within everyday social interactions of autistic and non-autistic people. The current study explored autistic adults’ camouflaging in an everyday social context via interpersonal process recall methodology (Kegan, 1969). A total of 17 autistic adults (8 females, 6 males and 3 agender/gender-neutral individuals) participated in a 10-min controlled social task designed to replicate a common day-to-day social situation. Participants then watched a video of their interaction with a researcher, actively identifying instances of camouflaging and discussing their experiences of camouflaging. Using thematic analysis, four themes were generated: (1) a strong desire for, yet uncertainty in, securing social acceptance and connection; (2) camouflaging, developed over time, as a means to achieve social acceptance and connection; (3) experiencing intrapersonal and interpersonal camouflaging consequences during social interactions; and (4) authentic socialising as an alternative to camouflaging. These findings are discussed with reference to the existing literature on stigma management outside the field of autism.

**Lay abstract:**

Many autistic people report that, despite personal costs, they use strategies to hide their autistic characteristics or appear non-autistic at work, school or university, when speaking with health professionals, or while socialising with certain friends and family members. These strategies are often referred to as camouflaging. This study explores camouflaging during everyday social interactions. A total of 17 autistic adults were filmed taking part in a common everyday social situation – a conversation with a stranger. They then watched the video of this conversation with a researcher and answered questions about camouflaging. These autistic people told us that they (1) had a strong desire to socialise with and be valued by other people but, because of negative past experiences, they often felt unsure about their ability to do so; (2) used camouflaging to help them to socialise and be valued by others; (3) experienced negative consequences when camouflaging (e.g. fatigue, anxiety and difficulties in friendships); and (4) sometimes socialised in more autistic ways instead of camouflaging. This study shows us how autistic people often change their behaviour because of the way they are treated by nonautistic people and that autistic people may benefit from programmes that help them to socialise in more authentically autistic ways, but only if their autistic social behaviour is met with understanding and acceptance from non-autistic people.

Autism spectrum disorder (henceforth ‘autism’) has historically been defined at the level of behaviours, identified via the discernible presence and absence of specific observable characteristics regarding social communication, as well as restricted and repetitive interests and behaviours (American Psychiatric Association, 2000, 2013). However, there is growing recognition that some autistic individuals appear behaviourally non-autistic in certain contexts. This phenomenon, termed ‘camouflaging’, has driven an emerging body of research and raised important questions regarding current diagnostic practices, intervention approaches and societal expectations for neurotypicality (e.g. Hull et al., 2017; Lai et al., 2017).

Defined as autistic individuals’ use of strategies to appear non-autistic and normatively socially competent during social interactions (Hull et al., 2017), camouflaging encompasses the complementary elements of hiding autistic characteristics (‘masking’; Hull et al., 2019) and employing strategies to overcome autistic cognitive difficulties/differences (‘compensation’; Livingston & Happé, 2017). In a recent online survey of 262 autistic adults, the majority reported that they consistently engage in camouflaging strategies in a range of everyday social situations, such as in interactions with work colleagues, friends, and health professionals (Cage & Troxell-Whitman, 2019). Camouflaging is associated with higher intelligence quotient (IQ) scores (Lai et al., 2017) and executive functioning abilities (Hull, Petrides & Mandy, 2020; Lai et al., 2017; Livingston et al., 2019); the female gender (Hull, Lai, et al., 2020; Lai et al., 2017; Schuck et al., 2019); and specific personality traits (Robinson et al., 2020). The precise mechanisms that enable camouflaging abilities, as well as the process through which these abilities are developed, are poorly understood. Yet, we know that camouflaging is linked to a range of negative consequences for autistic adults (e.g. misdiagnosis, identity confusion and mental health difficulties; Beck et al., 2020; Cassidy et al., 2018; Hull et al., 2017).

In considering approaches to investigating camouflaging, it is important to acknowledge that similar to other social phenomenon, camouflaging is not a construct located solely within an individual, rather it operates within social interactions that exist in a broader social context (Jaswal & Akhtar, 2019). As such, there is much to be gained by examining camouflaging via qualitative methods, with reference to the broader social context as well as other social phenomena and mechanisms operating in this context. One such social phenomenon, likely to be particularly relevant to the study of camouflaging, is the double empathy problem (Milton, 2012; Milton et al., 2018). The ‘double empathy problem’ suggests that due to differences in social norms and expectations, both autistic and non-autistic people experience communication, reciprocity and rapport problems during neurodiverse social interactions.

To date, several studies have provided valuable insights into the process, motivations and short- and long-term consequences of camouflaging via interviews and surveys of autistic adolescents and adults (e.g. Bargiela et al., 2016; Hull et al., 2017; Livingston et al., 2019b). Yet, traditional qualitative research techniques alone which rely solely on retrospective accounts often cannot yield the detailed and precise information required to develop a more comprehensive understanding of social phenomenon.

The present study aims to overcome these limitations through a qualitative investigation of camouflaging in an everyday social context via the novel use of interpersonal process recall (IPR: Kagan et al., 1969) methodology. While new to the field of autism, IPR has previously been used in psychotherapy, education, health and sport research to gain rich and detailed information about psychological experiences, processes and behaviours (e.g. Bartz, 1999; Burgess et al., 2013; Larsen et al., 2008; Marsh, 1983; Rhea et al., 1997). In the current study, participants first took part in a short introductory conversation with a non-autistic stranger. Following this interaction, they completed a semi-structured interview while viewing the audio-visual recording of their earlier social interaction. During the interview, participants actively identified specific camouflaging behaviours and processes and discussed their experiences of the experimental social interaction as well as their everyday social experiences more generally.

As described elsewhere (see Cook et al., 2020), participants described engaging in four main subtypes of camouflaging behaviours: masking, innocuous engagement, modelling neurotypical communication and active self-presentation. Masking behaviours concealed information about personal characteristics or circumstances and/or suppressed innate/autistic behaviours (e.g. limiting personal disclosures or suppressing hand movements). Innocuous engagement behaviours were conservative, passive and superficial social behaviours (e.g. smiling, mirroring or engaging in small talk). Modelling neurotypical communication behaviours involved altering verbal and non-verbal communication so as to conform with neurotypical conventions and preferences (e.g. altering facial expressions or gestures). Active self-presentation encompassed reciprocal, open and well-practised social behaviours (e.g. establishing and discussing points of similarity; providing elaborating information; or pre-planned or practised phrases, comments, questions or anecdotes).

The purpose of the current study is to detail the processes underlying these outward camouflaging behaviours and to capture the experience of camouflaging in autistic individuals during everyday social situations.

## Method

### Participants and recruitment

Participants were recruited via social media and through London-based autism support groups. Participants were eligible to take part if they met the following inclusion criteria: (1) aged above 18 years; (2) formally diagnosed with autism by an appropriate health care professional and/or multidisciplinary team; (3) without an intellectual disability (i.e. having an estimated IQ at/above 70); and (4) engaged in camouflaging (i.e. self-identifying as ‘engaging in camouflaging in their everyday lives’ and having a score of 100 or above on the Camouflaging Autistic Traits Questionnaire (CAT-Q); Hull et al., 2019). In total, 22 autistic individuals enrolled in the study but 1 did not meet the eligibility criteria, 1 withdrew before attending the lab, and 3 attended the lab but did not complete the full experimental procedure. Data for 17 adults (see Table 1) were collected in full and analysed.

**Table 1.** Participant demographics.

Pseudonym	Age range (years)	Gender	Ethnicity	CAT-Q
Angela	50–54	Female	White British	137
Ashley	40–44	Agender/gender neutral	White British	145
Beth	35–39	Female	White British	114
Belinda	30–34	Female	Mixed Other	138
Catherine	30–34	Female	White Other	130
Caroline	25–29	Female	Hispanic	106
David	45–49	Male	White British	113
Desi	45–49	Agender/gender-neutral	White Other	132
Edward	50–54	Male	White British	136
Eric	60–64	Male	White British	108
Fred	50–54	Male	White British	114
Frank	55–59	Male	White British	134
Greyson	20–24	Agender/gender-neutral	White British	148
Gail	50–54	Female	White British	160
Helena	55–59	Female	White British	158
Harriet	35–39	Female	White Other	162
Ian	55–59	Male	White British	121
	M = 44.53 SD = 12.03			M = 132.71 SD = 18.1

Precise ages are not provided to protect participant confidentiality. Mixed Other = mixed ethnicity other than Asian and White or Black and White; White Other = White ethnicity other than White British or Irish. CAT-Q: Camouflaging Autistic Traits Questionnaire (Hull et al., 2019).

All participants had estimated IQs above 70 (M = 112.47, SD = 4.65) on the Test of Premorbid Functioning (TOPF; Wechsler, 2009) and scored above the clinical screening cut-off of 26 (M = 39.71, SD = 6.02) on the Autism Spectrum Quotient (AQ; Baron-Cohen et al., 2001; Woodbury-Smith et al., 2005). All participants were diagnosed in adulthood and the mean age of diagnosis was 41.71 (SD = 12.18) years. The majority of participants were university educated, engaged in full or part-time employment or education, and lived independently (see Appendix 1 for supplementary education, employment and living arrangement information). Specific information on socio-economic status was not recorded.

### Measures and tasks

#### AQ

The AQ (Baron-Cohen et al., 2001) is a 50-item self-report measure of autistic characteristics. The AQ was used to give an estimation of autistic traits within the sample. Scores on the AQ range from 0 to 50 with higher scores indicating the presence of more autistic characteristics. Internal consistency in our sample was good (α = 0.81).

#### CAT-Q

The CAT-Q (Hull et al., 2019) is a 25-item self-report measure of camouflaging. Items are rated on a scale (from 1 = strongly disagree, to 7 = strongly agree) with higher scores indicating greater levels of camouflaging. A total CAT-Q score of 100 and above, was used to determine eligibility for the study. While the lowest end of this range indicated a relatively neutral endorsement of camouflaging behaviours, it was selected in an effort to avoid an overly prohibitive definition of camouflaging based on a newly developed measure. Internal consistency in our sample was good (α = 0.84).

#### TOPF – UK version

The TOPF (Wechsler, 2009) is a brief, standardised test of premorbid intellectual functioning suitable for individuals aged 16 to 90 years that involves reading 70 words aloud. The TOPF has been shown to accurately predict the Full-Scale Intelligence Quotient on the Wechsler Adult Intelligence Test – Fourth Edition for individuals with average and low average intellect (Watt et al., 2016). The TOPF also demonstrates good test–retest stability (r = 0.89–0.95; Wechsler, 2009).

#### Getting acquainted social task

Participants completed a standardised ‘getting acquainted’ social interaction modelled on prior research with non-autistic adults (e.g. Inderbitzen-Nolan et al., 2007; Plasencia et al., 2011; Taylor & Alden, 2010). This involved each participant partaking in a 10-min open-ended conversation with an unfamiliar female social partner. The experimenter (J.C.) explained to the participant that they would be spending time conversing with, and getting to know, this social partner and that they should act as they normally would when meeting a stranger whom they wish to make a good social impression on. The participant was then asked to enter the room where the social partner was waiting and to continue conversing with the social partner until the experimenter entered the room.

One non-autistic postgraduate psychology student acted as the ‘social partner’ during the task so as to replicate a degree of the double empathy problem (Milton, 2012) commonly faced by autistic people in everyday social contexts. In order to standardise the interactions and limit any potential distress or discomfort to participants related to the double empathy problem, she was trained to engage with participants in a friendly yet reserved manner following a protocol modelled on prior research (Inderbitzen-Nolan et al., 2007; Plasencia et al., 2011; Taylor & Alden, 2010). She was also aware all participants were autistic. Supplementary information on the training protocol is provided in Appendix 1.

One post-doctoral researcher served as an observer and checked the social partner’s adherence to the protocol and consistency across participants. The observer viewed audio-visual recordings of the social task and rated the social partner on the five dimensions of friendliness, talkative, disinterested, distant and self-disclosure using a seven-point scale (from 1 = not at all, to 7 = very much). Ratings were combined to give an overall rating of the social partner’s friendliness and warmth. This scale has previously been shown to have adequate internal consistency (α = 0.72; Plasencia et al., 2011). Mean warmth rating of the social partner was 27.82 (SD = 2.72), indicating she adhered to expected behaviour.

#### IPR interview

Immediately after the social task, participants completed a semi-structured interview with the experimenter based on IPR procedures (Larsen et al., 2008). The participant was informed that the purpose of the interview was to discuss ways in which they may have used camouflaging strategies during the experimental social task. The participant and experimenter then watched the audio-visual recording together and the participant was instructed to stop the video whenever they noticed themselves using, or thinking about, camouflaging strategies. When necessary, the experimenter asked the participant clarifying questions about their behaviour (i.e. to describe what they did or said). Once the behaviour was described, the experimenter followed the participants’ lead, asking follow-up questions about internal (e.g. their thoughts, emotions and motivations) and past experiences (e.g. how the participant learnt the behaviour) related to their behaviour.

Procedures for the ‘getting acquainted’ social task and IPR interview were carefully examined and modified by the research team (where necessary) to ensure suitability for use with, and accessibility for, autistic adults. Given that IPR interviews typically average two to three times the length of the preceding interpersonal interaction (Larsen et al., 2008), the length of the social task was restricted to 10 minutes to minimise the demands placed on the participants. In addition, the interviewer ensured participants clearly understood the purpose of viewing the video of the social task was to explore participants’ experiences related to camouflaging and not to judge or evaluate their social skills. The first four participants of the study provided open-ended feedback regarding the suitability and accessibility of the study procedure for autistic adults. No further modifications were required as a result of this feedback.

### Procedure

Ethical approval was obtained from the University College London Research Ethics Committee. Individuals who expressed interest in the study were provided with information sheets and given the opportunity to discuss these information sheets with the experimenter. Participants then provided their informed written consent and completed the demographic questionnaire, AQ and CAT-Q online. Participants who scored above 100 on the CAT-Q then attended the laboratory where they completed the social task, IPR interview and TOPF. Where available, participants brought written confirmation of their autism diagnosis to the laboratory to be verified by the experimenter (16/17 participants). In total, the testing session took approximately 90 min.

### Community involvement

Autistic people were involved in the current study as participants. They were not involved in the design or implementation of the study nor the analysis or dissemination of its findings. Wherever possible, the AASPIRE guidelines for conducting research with autistic participants were followed (Nicolaidis et al., 2019). Unfortunately, due to the unique IPR methodology used in the study, it was not possible to offer multi-modes of participation as suggested in these guidelines.

### Data analysis

Thematic analysis was conducted following the reflective thematic analysis approach developed by Braun and Clarke (2006, 2013, 2019; Terry et al., 2017). A critical realist framework was used to make sense of the data. As such, participants’ accounts were taken as being both true to them and mediated by features of the wider social context, and the impossibility of finding a decontextualised truth was acknowledged (Houston, 2001; Terry et al., 2017; Willig, 2013).

The data analysis process involved recursively moving through data familiarisation, coding, theme development and review. Analysis focused on identifying both semantic and latent meanings in the data following an inductive approach (Braun & Clarke, 2013). Analysis was led by J.C. but followed a collaborative approach with regular input at all stages from W.M. and L.C. Interview transcripts were read and re-read. Codes were devised, returned to and then revised. Codes were then grouped together to form candidate themes, and candidate themes were in turn reviewed and revised.

In reporting thematic analysis, it is important to situate the authors’ engagement with and positionality on the subject. None of the members of the research team are autistic. J.C., the lead author, is an autism researcher and practising clinical psychologist. She aligns more closely with a social model of disability over more medical and individualistic approaches (Shakespeare, 2006).

## Results

The four themes generated related to the experience of camouflaging for autistic people, including (1) a strong desire for, yet uncertainty in, securing social acceptance and connection; (2) camouflaging, developed over time, as a means to achieve social acceptance and connection; (3) experiencing intrapersonal and interpersonal camouflaging consequences during social interactions; and (4) authentic socialising as an alternative to camouflaging.

### Acceptance and connection: ‘[Autistic people] often genuinely want to make a connection they just find it difficult’

Participants were motivated to interact with others in a manner that facilitated social acceptance and connection but held doubts about their ability to do so. Participants reflected on the need to create a particular kind of impression to be ‘valued’ and ‘liked’ by others. In turn, they felt this would increase the likelihood of much desired future social interaction and ultimately ongoing companionship. Participants felt that managing their impression was particularly important during initial interactions with ‘new’ people, suggesting unfamiliar social partners were more likely to hold them in negative regard.

Some participants sought to promote their social image via positive attributes, reporting attempts to be perceived as ‘similar’ to their social partner, ‘friendly’, ‘nice’ and ‘intelligent’. However, other participants spoke of the need to defend their social image against potential negative social evaluations. These participants focused their impression management efforts on avoiding negative attributes and ensuring that they were not perceived as, for example, ‘weird’, ‘strange’, ‘threatening’, ‘dominating’ or ‘boring’.

Some participants’ efforts were focused on concealing their autistic identity or portraying a non-autistic, conventional or otherwise valued identity. When reflecting on her behaviour during the experimental social task, Helena positioned her autistic identity as one that must be hidden in order to present as possessing the more valued, non-autistic identity: ‘This is very safe ground . . .. because, it gives a little bit about me away but not enough . . . it’s nothing that really indicates that maybe I’m high functioning autism or anything like that’. Similarly, Fred highlighted his effort to position himself as possessing a valued or desirable identity: ‘I suppose I’m trying to say that I am a responsible member of society in some way’.

Some participants sought to create a desirable social impression by engaging in neurotypical, as opposed to autistic, social behaviours. Some participants engaged in these behaviours during the experimental social task despite assuming the research assistant knew of their autistic identity. Thus, they appeared to believe that interacting in accordance with non-autistic social norms and expectations was required to gain acceptance during social interactions even when their autistic identity was known. This requirement was articulated by Catherine when explaining her use of hand-wringing to reduce anxiety instead of hand flapping: ‘This [demonstrates hand flapping], works a lot better but it gets people’s attention a lot more so we don’t this [hand flapping], we do this [demonstrates hand-wringing], it’s a lot more socially acceptable’.

Past experiences of criticism, rejection and misunderstanding during social interactions were central in participants’ efforts to interact with others in a manner that facilitated acceptance from, and social connection with, others. Participants described experiences in which non-autistic social partners explicitly or implicitly associated participants’ displays of overt autistic behaviours with negative social traits, for example, being ‘rude’, ‘sick’ or ‘shifty’:

You’re stupid, you’re abnormal or when you start to do this [demonstrates body rocking] it’s a sign that you’re sick, something is wrong in your brain and I have heard that from my own father when I was little. (Harriet)

Participants also positioned themselves as responsible for the outcome of past negative social experiences with non-autistic others, attributing interpersonal difficulty or rejection to failures in their own interpersonal behaviour and self-presentation:

When [I] went to the toddler groups and I thought I can’t talk to these women. I don’t know what to say. They would all say, ‘Oh, we’re having problems with so and so’s eating or sleeping’, so I’d come back the next week and I’d have articles and books and have loads of suggestions. This might work. And they did, they were working [for] me. And of course, they all hated me and they said you’re a know it all. And [I] was like no, I think I know nothing, that’s why I read the books. (Gail)

A lifetime of such social experiences appeared to leave some participants uncertain and anxious about their ability to successfully portray the kinds of social impressions that would lead others to value social relationships with them. Camouflaging was seen by participants as a means of improving their social impression.

### Camouflaging process: ‘it would be to appear non-autistic, that is the main reason why I personally do that’

Participants reported engaging in social behaviours that demonstrated their positive attributes and highlighted similarities between themselves and their social partner. They avoided behaviours that potentially signalled undesirable traits; fostered conflict; or created anger, discomfort or distress in others:

I brought up global warming, but I thought to myself, ‘No don’t bring up global warming, don’t start talking about that’. I think it’s a bit of a sensitive topic which some people believe, some people don’t believe, and in a way sometimes it can cause an argument. (Caroline)

Some participants described displaying verbal and non-verbal behaviours perceived to be associated with non-autistic socialising while suppressing their more innate (and often autistic) verbal and non-verbal behaviours: ‘What I’m trying to do is to smooth my tone of voice out . . . and make it sound less choppy which seems closer to what most neurotypical people do’ (Greyson).

Some participants selectively shared information about themselves, emphasising their more normative interests, and characteristics, or circumstances and minimising more autistic or less conventional interests, characteristics and difficulties: ‘I guess I’m acutely aware of [autistic] blokes that are like “I like trains. I like buses,” and I don’t want to be seen like that you know?’ (Beth).

The camouflaging process appeared to assist some participants to compensate for personal difficulties that interfered with their ability to adhere to non-autistic social norms. Participants identified experiencing challenges with, for example, understanding others’ perspectives, reading subtle social cues, processing verbal information quickly and remembering faces as well as an awareness of the manner in which these challenges affected particular aspects of social interactions: ‘I find that difficult. You know, whether too little or too much information. What the information that person wants or if it’s just small talk at face value’ (Ian).

Behaviours exhibited or suppressed by participants functioned as a part of their idiosyncratic solutions to these problems. For example, Desi described being aware that they had difficulty maintaining conversations and used a scripted phrase to overcome this: ‘I did my usual party trick of she asks me a question and I just flip it back and I give her answers and flip it back and say, “And you?” It’s my way of keeping the conversation going’.

The camouflaging process also appeared to involve the dynamic monitoring of, and adaption to, cues in the social environment. Participants spoke of ‘constantly’ monitoring their own social behaviour to ensure they adequately performed camouflaging behaviours. At the same time, they described closely examining their social partner’s interpersonal cues for signs of, for example, engagement and interest or boredom and discomfort. They then adjusted their behaviours in response to these cues:

[The social partner] is nodding and appears to be engaged which is why I carried on with conversation. If she started to look bored and not terribly interested, I would have gone to a different topic of conversation, probably her. (Eric)

Signs of camouflaging ‘failure’ appeared to be particularly salient to participants when monitoring their social performance. Some participants spoke about failing to achieve their camouflaging or self-presentation goals: ‘I don’t look as normal as I think I do’ (Belinda). Other participants identified instances of themselves failing to keep certain autistic characteristics ‘under control’, perform specific camouflaging behaviours or read and respond to their social partner’s social cues: ‘I kept thinking I shouldn’t really wriggle my legs so much, but I just couldn’t help it’ (Desi).

Participants’ idiosyncratic repertoires of camouflaging behaviours were developed and refined through an iterative process over time. Some participants spoke of learning new behaviours or changing their behaviours in response to criticism, rejection or devaluation from non-autistic others: ‘Someone said to me, you never make eye contact, you look really shifty. So, I had to train myself to do eye contact’ (Angela).

Other participants described carefully observing people (autistic and non-autistic) engaging in social interactions from afar, carefully noting the manner in which they engaged with and responded to each other. Some reported focusing in particular on the behaviours of socially valued individuals and of trialling these behaviours in their own social interactions:

I used to hate her laugh because it used to give me a headache but everyone seemed to really like her and they always used to say things like, ‘Oh, she’s so happy, she’s so funny’, and I thought, ‘Oh, maybe I will try and make myself a bit more like her’. So I changed my laugh and I started practising my laugh to make it a bit more like hers. (Ashley)

Some participants reflected that while many of these behaviours initially required much effort and conscious thought, after many years, certain camouflaging behaviours now occurred automatically or unconsciously. However, other participants did not experience any automatisation of their camouflaging behaviours.

### Intrapersonal and interpersonal consequences of camouflaging: ‘it’s a lot more taxing, it’s a lot more difficult and it’s a lot less authentic’

When reflecting on their experience of the experimental social task, some participants identified multiple, discrete episodes of increased anxiety. These episodes were often triggered by threats to their self-presentation goals, for example, participants becoming aware of social cues indicating the social partner may be criticising, rejecting, misunderstanding or otherwise devaluing the participant or participants’ uncertainty regarding how to act or respond:

I mean [the social partner] is quite uncomfortable there. And I can sense that she’s covering herself and fiddling and I am sort of thinking, ‘Oh God, how can I make her feel more comfortable?’ But I don’t really know what I’m going do, but I’m worried. (David)

The camouflaging process was also viewed by participants as cognitively taxing, exhausting and difficult to sustain. Some participants identified specific camouflaging behaviours as being particularly effortful or challenging:

I do make eye contact with people but you can see it is reduced here because, and that is generally where it is reduced, I tend to look away because I’ve got to think about what I’m thinking about and trying to look at someone at the same time is extra burden. (Frank)

Other participants described cognitive aspects of camouflaging such as monitoring their performance and the social cues of others as being challenging and energy consuming:

I think all of those things that go on in the background can be quite exhausting for someone of the spectrum because you are managing all that stuff whereas for another person it’s just a natural back and forth thing. Whereas you have to manage your thoughts of, ‘Am I talking too much, or just talking too much and not realising?’ and it’s just all the things. (Catherine)

Participants’ descriptions of their social experiences suggested they continued to experience social cognition difficulties while engaging in camouflaging. Some participants spoke of being unsure about what to do or say during interactions and of finding it difficult to read others’ social cues: ‘I, as with most people with Asperger’s/autism am not very good at interpreting body language, so I tend to feel–am I going on about something and the other person is just bored?’ (Edward)

The effort required to successfully overcome these social difficulties and simultaneously camouflage contributed to participants experiences of exhaustion and fatigue during social interactions. In the same way, the uncertainty caused by social confusion and insecurity appeared to contribute to participants’ feelings of anxiety.

Camouflaging appeared to have additional interpersonal consequences for participants’ social interactions. Some participants paradoxically described camouflaging as interfering with their ability to fully engage and effectively communicate during social interactions and in turn make certain desired impressions. For example, some participants associated the performance or concealment of particular behaviours with exacerbations in receptive and expressive language difficulties. Angela explained how engaging in the camouflaging behaviour of eye contact interfered with her capacity to express herself and potentially, her ability to portray an impression of competence:

If I am trying to make a good impression with you I have two options. I carry on looking at you and then have less brain function and so I will not be able to answer your question or I will have less ability to process what you are saying. If I look away I can listen more and I can think more. So although in an interview or whatever where I am trying to pretend to not be autistic I think I would have to allow myself- I would make some eye contact as much as possible but I have to allow myself [to not make eye contact] otherwise I am just going to end up talking gibberish.

Despite being viewed as necessary to develop much desired social connections, some participants also described camouflaging as limiting the closeness and intimacy of their social relationships:

It’s a lot less authentic because you’re [non-autistic people] being yourself in a different mood versus [autistic people] being someone else entirely and if in all your close relationships you are pretending to be someone else then even if you superficially seem to have a really good social life, you have no genuine relationships with anyone because none of them really know you. (Greyson)

### Authentic socialising: ‘I am not ashamed anymore and I am feeling I have the right to express the ideas in the way I want to express them’

A diminishing desire to socialise in accordance with non-autistic norms or present a non-autistic identity was described by some participants. Often these participants reported consequent reductions in the frequency of their camouflaging during everyday social interactions. However, this experience was more complicated for some participants who spoke of difficulties engaging in, or even ‘knowing’, alternative means of socialising after a lifetime of camouflaging:

But I don’t want to anymore. I’ve had enough of it. I want to switch it off now. I am fed up with it. I don’t feel like it’s got me . . . I feel it is more for other people’s benefit than for my benefit and I feel like it takes up so much time and energy that I need to be able to switch it off but I feel like I have been doing it for so long that I don’t know how. (Ashley)

Engagement in more authentic socialising was associated with participants’ growing understanding and acceptance of themselves and in particular their social needs:

Now I am more confident of who I am and why I reacted like that. I’m tending to camouflage less because I am not ashamed anymore and I am feeling I have the right to express the ideas in the way I want to express them so if I want to move a bit because it is helping my cognitive flow or if I want to not look in the eyes I’m not going to anymore because that is very damaging in the past. (Harriet)

For some participants, the diagnostic process was central to the development of their awareness and self-acceptance such that it provided them with both recognition and validation of their social differences and needs:

I think I do it less than I used to because now I don’t have to pass as NT [neurotypical] do I? I’ve got a diagnosis, whereas before, why can’t I be normal? Something wrong, something not working. Just be normal. (Desi)

Some participants reflected on the role of others’ understanding and acceptance in creating a ‘comfortable’ and ‘safe’ environment that enabled authentic socialising. In this regard, familiarity with autism was framed as key with participants saying they camouflaged less in interactions with other autistic people or non-autistic people whom were perceived to be knowledgeable about autism. Non-judgemental and welcoming attitudes towards diverse interpersonal styles as well as diversity more generally were also described as important. In this way, participants’ friends and partners were often positioned as being both knowledgeable about and accepting of idiosyncratic or autistic difference: ‘I can trust them not to react badly and not to decide that they don’t like me and treat me badly because of that’ (Greyson).

Within these contexts, participants described enacting a more autistic interpersonal style by engaging in more overtly autistic body movements, levels of reciprocation and conversational exchanges: ‘When I get very excited and if I am around people who I trust that it is ok to do that [hand flapping]’ (Catherine). Participants also spoke of being empowered to communicate their social difficulties and differences to others as well as any adaptations they required: ‘I will say to them, I’m listening to you just . . . I might not be looking at you’ (Beth).

More autistic socialising appeared to be associated with increased feelings of ease, authenticity, enjoyment and decreased anxiety, stress, and exhaustion. This was articulated by Harriet, in her explanation of her mental state after engaging in body rocking and other stimming movements throughout the day: ‘It’s making it beneficial for me just going through the day and arriving at the end of the day and not being overwhelmed because during the day I was reliving the pressure’.

## Discussion

For the first time in autism research, we used a combination of IPR methodology and thematic analysis to explore autistic adults’ experiences of camouflaging. Taken together, the four themes generated here detail the development, process and consequences of camouflaging for our participants. Participants commonly encountered negative social experiences and responses from others as a result of their autistic characteristics and behaviours. Driven by their need for social connection, participants attempted to systemise the social environment and augment these social experiences and responses. Over time, they developed a belief that they must change their interpersonal presentation in order to achieve acceptance and connection as well as an ability to do so – the ability to camouflage. Their belief is activated in particular social contexts leading them to engage in a dynamic camouflaging process involving exhibiting behaviours consistent with non-autistic identity and norms; monitoring personal social performance; and evaluating other’s interpersonal cues. Engagement in the camouflaging process results in situ intrapersonal and interpersonal consequences.

### Camouflaging, social motivation and mutual social influence

Participants expressed a strong interest in, and motivation towards, interacting with others in a manner that facilitated social connection and further interaction. Such evidence of social motivation among autistic adults is consistent with past camouflaging research (e.g. Hull et al., 2017; Livingston et al., 2019a). It also challenges the social motivation theory of autism (Chevallier, Kohls, et al., 2012), providing further evidence that social motivation is not universally diminished among autistic people (e.g. Jaswal & Akhtar, 2019).

Related to the concept of social motivation, mutual social influence refers to the tendency of individuals to influence, and be influenced by, their social environment (Forgeot d’Arc & Soulières, 2019). Participants’ accounts emphasised the role of mutual social influence in camouflaging such that they sought to manage others’ perceptions of them by portraying a non-autistic social presentation because, based on their past social experiences, they believed doing so would lead others to value social interaction and relationships with them. Such accounts support existing qualitative and experimental research from across the lifespan demonstrating autistic people are suspectable to social desirability effects (Gernsbacher et al., 2019), experience reputation concerns (e.g. Bargiela et al., 2016; Cage et al., 2016a; Hull et al., 2017) and engage in reputation management or strategic self-presentation (Cage et al., 2013, 2016b; Scheeren et al., 2016, although see Chevallier, Molesworth, & Happé, 2012; Izuma et al., 2011).

### Camouflaging and stigma

Negative or difficult social encounters with non-autistic others were often described by participants. As outlined in the double empathy problem (Milton, 2012; Milton et al., 2018) due to the differences in social norms and expectations, both autistic and non-autistic people experience communication, reciprocity and rapport problems during neurodiverse social interactions. However, research suggests autistic people also experience devaluation, rejection and misunderstanding related to their autism label and/or overt autism-related behaviours (e.g. Kinnear et al., 2016; Milton, 2012; Milton et al., 2018; Sasson et al., 2017; Sasson & Morrison, 2019). Indeed, some scholars suggest autistic people represent an identity-based minority group subjected to social stigma and disadvantaged social status (Botha & Frost, 2020). As such, our participants reported attempts to gain acceptance and social connection by presenting and interacting in line with non-autistic identity and norms and is consistent with broader research on stigma management.

Individuals with concealable stigmas (e.g. mental illness diagnosis, particular sexual orientations or a history of incarceration; Goffman, 1963; Jones et al., 1984) use identity or impression management strategies to control their interactions with others in order to conceal their stigmatised identity and pass as a more valued identity, thereby securing the acceptance and belonging of others (e.g. Goffman, 1963; Leary, 1999; Olney & Brockelman, 2003). Such strategies can include changes to interpersonal behaviour (e.g. tone of voice, gestures or posture; Pachankis & Goldfried, 2006) but predominately involve controlling potentially exposing information via deception, concealment and evasion (Clair et al., 2005; Herek, 1996) as well as close monitoring of personal behaviour and the behaviour of others (Olney & Brockelman, 2003; Pachankis, 2007).

In a similar manner to other stigmatised identities (e.g. mental illness; Quinn et al., 2004), autism could be conceptualised as existing on a continuum from conspicuous to concealable, depending on an individual’s particular profile of autistic behaviours as well as their ability to conceal these behaviours. In this way, camouflaging may be thought of as a form of stigma management that is available to autistic individuals with more ‘concealable’ autism (Cage & Troxell-Whitman, 2019). Indeed, the camouflaging process described by our participants represents a dynamic and sophisticated means of influencing and shaping the social environment that bears resemblance to the repertoire of behaviours described in the stigma management literature (e.g. Clair et al., 2005; Olney & Brockelman, 2003; Pachankis & Goldfried, 2006). Participants actively adapted their interpersonal behaviours, selectively disclosed personal information and engaged in performance and impression monitoring. However, changes to interpersonal behaviours were more central to participants’ accounts of camouflaging than selective disclosure, omission and concealment of personal information. This differential emphasis reflects the unique manner in which autistic behaviours attract as much or more stigma as an autism diagnosis. It also likely reflects non-autistic people’s difficulties understanding autistic social communication (Crompton et al., 2020; Edey et al., 2016; Sheppard et al., 2016) and the consequent need experienced by autistic people to change their social behaviour and presentation so as to facilitate effective communication during neurodiverse social encounters (Milton, 2012; Milton et al., 2018). In further contrast, participants highlighted the role of camouflaging in managing autistic differences/difficulties that lead to breakdowns in the impression management process and/or hindered effective communication with non-autistic others. As such, in the case of autism, camouflaging may represent both a means of portraying a valued social identity and overcoming communication difficulties in neurodivergent socialising.

### Consequences of camouflaging

In line with previous qualitative research (e.g. Hull et al., 2017; Livingston et al., 2019b), participants associated camouflaging with adverse in situ consequences. Specific camouflaging strategies and components were identified by participants as being difficult or taxing to perform. Feelings of anxiety while camouflaging were similarly common and often triggered during the experimental social task by perceived threats to participants’ self-presentation goals. Furthermore, camouflaging was paradoxically described as interfering with participants’ ability to fully engage and effectively communicate during social interactions; make certain desired impressions; and limiting authenticity and closeness within social relationships.

A dearth of experimental research exists examining the impact of camouflaging for autistic individuals with regard to cognitive resources; achievement of camouflaging and other interpersonal goals; and satisfaction in social relationships. Emerging quantitative research examining associations between camouflaging and anxiety (e.g. Cage & Troxell-Whitman, 2019; Lai et al., 2017; Schuck et al., 2019) have yielded inconsistent results. Further research is thus required to better delineate the relationship between camouflaging and anxiety.

The negative intrapersonal and interpersonal consequences of camouflaging described by our participants are consistent with experimental research on stigma management. Experimental research suggests that actively concealing stigma during social interactions decreases cognitive resources (Critcher & Ferguson, 2014; Smart & Wegner, 1999) and increases emotional strain (Barreto et al., 2006). Concealment of stigma is also associated with reduced feelings of belonging, authenticity and non-stigma-related self-disclosure, as well as less positive observer rated social performance (Newheiser & Barreto, 2014). The psychological distress, cognitive burden and interpersonal costs associated with camouflaging may be similar in nature to that of stigma management in other concealable stigmas (e.g. mental illness diagnosis, minority sexual orientation or low social class background). However, given that participants described experiencing persistent social cognition difficulties during social interactions, we hypothesise that the adverse consequences of stigma management are likely exacerbated in the case of autism.

In the current study, participants associated more authentically autistic socialising – that is, engaging in more overtly autistic social behaviours, explaining autistic social differences and communicating autistic social needs – with decreased negative affect as well as increased positive affect. Participants’ accounts highlighted the role of both their own and others’ awareness and acceptance of diversity and autism in facilitating more authentic socialising. Such findings are in line with research suggesting disclosing a stigmatised identity in a supportive environment may elicit multiple benefits including increased self-esteem and decreased distress (Corrigan & Matthews, 2003), increased likelihood of receiving social support (Beals et al., 2009) and improved social interactions (Newheiser & Barreto, 2014). However, at present, more authentic socialising may not be associated with improved psychological well-being for the majority of the autistic community who lack access to such supportive environments (Botha & Frost, 2020).

### Clinical implications

Insights gained from the participants in the current study have important clinical implications. Formal autism interventions explicitly teaching, for example, non-autistic social behaviours, may have the un-intended consequence of explicitly or implicitly reinforcing the notion that autistic people need to present and interact in line with non-autistic expectations and norms in order to be accepted and valued by society, and in turn, encouraging camouflaging (e.g. Bottema-Beutel et al., 2018). Interventions that assist autistic people to understand and accept their social differences, as well as an ability to communicate these differences, may improve the everyday social experiences of autistic people. In order for autistic people to benefit from authentic socialising though, their autistic social behaviour must be met with understanding and acceptance on the part of non-autistic social partners.

While the role of camouflaging in conforming to non-autistic social expectations was emphasised by participants, so too was the role of camouflaging in overcoming communication challenges in neurodivergent socialising. Of significance, participants highlighted the manner in which camouflaging assisted them to overcome difficulties in identifying and interpreting non-autistic verbal and non-verbal behaviours; understanding the rationale for or intentions behind non-autistic social behaviours; and maintaining social coordination with non-autistic people. These experiences highlight the manner in which the social difficulties of camouflagers are often overlooked (e.g. Bargiela et al., 2016; Hull et al., 2017). Thus, it is important to acknowledge the role of interventions that assist autistic people to understand non-autistic social behaviour, and vice versa non-autistic people to understand autistic social behaviour, in improving neurodivergent social communication. However, it is equally important to acknowledge the effectiveness of autistic peer-to-peer social communication (Crompton et al., 2020).

### Strengths and limitations

The results of the current study are strengthened by its novel methodology. Via the use of a standardised social task, involving a non-autistic social partner we successfully re-created a quasi-everyday social situation in which autistic people may be motivated to camouflage. IPR interviews yielded in-depth information about autistic adults’ motivations, cognitions and emotions related to camouflaging not before generated by more traditional qualitative research methods.

It is important to acknowledge that the themes generated here reflect the specific experiences of a sample of verbally fluent, late diagnosed, adults who self-identified as engaging in camouflaging. Camouflaging may be particularly pivotal in the lives of late diagnosed autistic people and in this regard, the current study provides valuable insights into the often under-researched experiences of this group. Nonetheless, future research is needed involving, for example, young adults, early diagnosed individuals or those with an intellectual disability, for whom experiences of camouflaging may differ.

## Conclusion

The four themes reported here detail the manner in which our participants developed camouflaging through an iterative process over time in order to overcome barriers to social acceptance and connection and capture their experience of engaging in camouflaging and authentic socialising during interpersonal interactions. Our findings suggest the non-autistic majority’s understanding and interpretation of autistic behaviour impacts on autistic people’s beliefs about themselves and the social world and in turn, the manner in which they engage in social interactions. Our findings resonate with research on concealable stigma while also suggesting potential differences in the function and consequence of identity management and camouflaging. These insights add to the growing recognition of the need for innovative, systemic approaches for improving the quality of social experiences for neurodivergent people.

## Appendix 1

### Supplementary methods

#### Participant characteristics

Additional participant characteristics are provided in Table 2.

**Table 2.** Education, employment and living circumstances.

	*n* (%)
Highest level of education achieved	
PhD	1 (5.8)
Master’s degree	7 (41.2)
Bachelor’s degree	8 (44)
A-levels (school leaving qualification)	1 (5.8)
Current day activity (categories not mutually exclusive)	
Working full-time	6 (35.5)
Working part-time	7 (41.2)
Voluntary employment	2 (11.8)
Caring duties	1 (5.8)
Student	4 (23.5)
Unknown	1 (5.9)
Current living circumstances	
At home with partner/children	8 (47.0)
At home alone	8 (47.0)
At home with flatmates/friends	1 (5.8)

Percentage may not sum 100% because of rounding.

### Measures and tasks

#### Social partner training protocol

Through a series of practice role-plays, the post-graduate psychology student was trained to consistently and naturally (1) speak in a warm tone; (2) allow 2- to 3-second pauses after the participants’ last comment before speaking; (3) allow a 10-second pause in the case of non-reciprocation (i.e. if she asked two questions in a row or made two comments in a row and the participant minimally reciprocated); (4) occasionally offer encouraging comments (e.g. ‘Tell me more about that’); (5) engage in a moderate level of self-disclosure; (6) engage in a moderate number of minimal encouragers; and (7) maintain steady and comfortable eye contact while looking away briefly at times. She was given a list of conversation topics to discuss in order to maintain consistency across participants.
